# Cross-Sectional and Longitudinal Associations Among Children’s Interpersonal Trust, Reputation for Trustworthiness, and Relationship Closeness

**DOI:** 10.3389/fpsyg.2021.634540

**Published:** 2021-10-01

**Authors:** Qinggong Li, Zhuocheng Li, Wenyu Zhang, Yang Wang, Gail D. Heyman

**Affiliations:** ^1^Department of Psychology, Zhejiang Normal University, Jinhua, China; ^2^Department of Psychology, University of California, San Diego, San Diego, CA, United States

**Keywords:** socialization, personal relationship, children, interpersonal trust, social reputation

## Abstract

Interpersonal trust plays a crucial role in the formation and maintenance of social relationships. The present cross-sectional and longitudinal research examines the development of interpersonal trust judgments with reference to (1) the trustee’s reputation for trustworthiness, and (2) the nature of the trustor’s relationship closeness with the trustee. There were 194 7- to 13-year-olds who participated in the first wave of the study, and 107 of those individuals also participated in two subsequent waves across a 2-year period. Both cross-sectional and longitudinal results showed that with age, reputation for trustworthiness becomes less important and relationship closeness become more important. We also found that relationship closeness played a greater role in interpersonal trust evaluations for girls than for boys. These findings indicate that the way children make trust evaluations becomes increasingly relationship-specific over time and is more relationship-specific for girls than for boys.

## Introduction

The human capacity to cooperate and collaborate with one another greatly extends what we are capable of accomplishing as individuals and as a species (Tomasello and Hamann, 2012). This capacity allows us to form non-kin relationships that meet our social and emotional needs, learn from the successes and failures of others, and carry out projects that are far too complex for a single person to complete. However, working with others entails risk, as can be seen when we count on people who fail to follow through on their commitments or do not have our interests at heart. In this way, trust can be viewed as a willingness to make oneself vulnerable to the actions of another individual based on the expectation that that individual will perform actions that are important to him or her (Mayer et al., 1995).

Most developmental research on person-specific trust has examined how children assess whether others are reliable sources of information (Harris, 2007; Heyman, 2008; Gelman, 2009; Mills, 2013). This work suggests that during the preschool years, children learn to use a variety of cues to judge the extent to which individuals can be trusted to provide accurate information about word labels and other facts. These cues include a source’s history of prior accuracy (Koenig and Harris, 2005; Jaswal and Neely, 2006; Pasquini et al., 2007; Birch et al., 2008; Corriveau and Harris, 2009; Poulin-Dubois and Chow, 2009), expertise (Landrum et al., 2013; Boseovski and Thurman, 2014; Lane and Harris, 2015), benevolence (Landrum et al., 2013; Lane et al., 2013), honesty (Lane et al., 2013; Li et al., 2014), familiarity (Corriveau and Harris, 2009), gender (Taylor, 2013), and level of agreement with others (Corriveau et al., 2009).

Research assessing when and how children come to view specific individuals as reliable sources of information deeply informs our understanding of how they evaluate information and learn from others. However, this research does not clearly speak to theoretical accounts of trust that focus on the centrality of vulnerability by placing an emphasis on a willingness to take personally meaningful risks (Ring and Van de Ven, 1992; Sitkin and Roth, 1993; Mayer et al., 1995; Hall et al., 2001; Bos et al., 2002). This is because the social and emotional stakes for children within such contexts are often minimal or unclear. For example, if a child learns the wrong name for a novel object as a result of trusting an unreliable informant, the problem is likely to be easily remedied once the child is corrected, and there is minimal risk that anything of importance will be lost.

There has been a greater focus on the issue of vulnerability in research examining trust among adults (Deutsch, 1958; Johnson-George and Swap, 1982; Lewicki and Bunker, 1995; Hall et al., 2001; Bos et al., 2002; Colquitt et al., 2007). This literature has established that interpersonal trust typically develops over time and can easily be damaged by behavior that undermines cooperation (Lewicki and Bunker, 1995).

There has also been some developmental research examining children’s trust in relation to vulnerability (Betts and Rotenberg, 2008; Xu et al., 2013; Betts et al., 2014; Fu et al., 2015). This research typically includes measures in which participants are asked to report the willingness to trust on specific individuals in contexts that might leave them vulnerable. For example, Xu et al. (2013) found 7- to 11-year-olds considered both honesty and benevolence when making trust judgments (such as promise-fulfillment, secrets-sharing and information-seeking), and older children were more likely than younger children to focus on benevolence other than honesty.

Interpersonal trust is typically conceptualized as being reciprocal within dyadic interactions (Rotenberg et al., 2008). This focus is closely tied to developmental conceptualizations of friendship in which mutual trust and intimacy become more central components of friendship as children get older (Damon, 1983). Positive peer relationship can promote children’s social adaptation, and help children better understand others and society (Hartup, 1996). Moreover, positive peer relationship can facilitate children’s communication with others, which may increase the closeness of the relationship with others. During peer interaction, children can deepen their understanding about others, which helps to cultivate their patience and care for other, and thus promote peer acceptance (Oberle et al., 2010).

However, it is also possible to develop trust outside of dyadic contexts, and to decide whether to trust others based on their general reputations, much as people decide whether to trust politicians they have never met before. This possibility is also in line with recent theoretical work showing that young children are concerned about how they will generally be viewed by others (Shaw et al., 2014; Rapp et al., 2019). There is suggestive evidence that children use both of these frameworks when making trust assessments. When children ages 6 and older make determinations about the relative trustworthiness of their peers, they take into account both information about the extent to which trustees are perceived to have a reputation for being trustworthy, and the nature of their personal relationship with the trustee [see Betts and Rotenberg (2008) and Betts et al. (2014)]. Trustworthiness is also the foundation of friendship, which can only be formed and strengthened when both friends trust each other. Trustworthiness is one of the characteristics of friendship once it is established (Mcauley et al., 2012). Based on the three-stage friendship development model (Damon, 1983), 8- to 10-year-old children are most likely to form friendships based on trust, though their sense of companionship becomes more complex by this time, recognizing that people who can help in times of need are regarded as friends. When they grow up, children would understand the value of trustworthiness more, and that trustworthiness could facilitate their friendships (Xu et al., 2013).

According to Mayer et al. (1995), we define the children’s interpersonal trust as a willingness to make oneself vulnerable to others in a social interaction with personally meaningful risk. In addition to our measure of interpersonal trust, we also assessed relationship closeness and peers’ reputation for trustworthiness. We used both cross-sectional and longitudinal methods to go beyond a typical “snapshot” view of trust to inform models of how trust develops over time within the context of social interaction (Lewicki et al., 2006). Using both methods also allows us to rule out some possible artifacts, like cohort effects, when interpreting our findings.

In addition to our central research question, we were also interested in whether the extent to which trust was viewed as relationship-specific might differ as a function of gender. It is plausible that they may, given previous work suggesting that gender can sometimes affect selective trust (Taylor, 2013) and that there are gender differences in relationship qualities (Benenson and Schinazi, 2004; Watson, 2012). For example, girls are more likely to meet their closeness needs through friendship (Hall, 2011). Moreover, studies have shown that there exists a gender difference in trustworthiness in older children: peer reports suggest that girls are more trustworthy than boys (Rotenberg et al., 2004). The difference between boys and girls may be explained by the fact that girls are socialized to have close peer relationships, while boys are not (Berndt and Perry, 1986), and the consequent need for trustworthy behavior for girls to participate in these relationships.

To sum up, the first aim of this study is to examine how factors associated with personal-specific interpersonal trust, such as interpersonal closeness and reputation for trustworthiness, shape children’s trust judgments and how they might change with development in Chinese culture. As Chinese children grow, they might increasingly share what seems to be the general adult view that trust has to be viewed in terms of specific relationships (Peng, 1998). Specifically, we predicted that over time children would move away from focusing on someone’s general reputation for showing trustworthiness to concentrating on the degree to which individuals demonstrate trustworthiness within specific relationships. The second aim is to explore whether there are gender differences in children’s propensity to make trust judgments from the perspective of development, particularly when social cues such as interpersonal intimacy and peer’s reputation for trustworthiness are considered. We predicted girls would value interpersonal closeness more than boys in assessment of trust, because girls may be more sensitive to creating, maintaining and destroying interpersonal relationships.

## Materials and Methods

### Participants

A total of 194 children were initially recruited from an elementary school in Jinhua, a medium-sized city in China, and all of them were from the Han ethnic group. The highest level of education for the participants’ fathers was 30% middle school, 40% high school, 29% university, and 1% unknown, and for the mothers it was 23% middle school, 44% high school, 32% university, and 1% unknown. Self-reported family income was 75% average income, 11% significantly higher than average income, 13% significantly lower than average income, and 1% unknown. The study was approved by the University Research Ethics Committee and all children who gave their oral assent to participate and had consent from a parent or legal guardian were included in the research.

There were three waves of testing across a 2-year period. All of the participants were assessed for the first time in November 2012. Sixteen participants were excluded from the data analyses, because they chose not to complete the study (*N* = 6) or because they gave the same responses to all questions (*N* = 10). Each participant would give from 111 to 153 rating in our study. Our assumption is that if a child gives identical ratings on more than 100 questions, he or she probably was not taking the task seriously. This resulted in a final sample of 178 children (84 girls; mean age = 9.92 years, SD = 1.58, age range = 7–13 years) for the first wave of testing. We chose the age range of 7–13 years for the first wave because the children had been classmates for at least 1 year. The existing literature shows that 1 year is sufficient for children to get know each other well in a classroom setting (Betts and Rotenberg, 2008).

A subsample of 107 children from the original sample was also assessed in both November of 2013 and November of 2014. This subsample included all of the children who had been expected to be at school after 2 years from Time 1. Nine of these participants were excluded from the data analyses because they did not complete the next two waves of the study (*N* = 5), or because they gave the same response to all questions (*N* = 4). This resulted in a final subsample of 98 children (40 girls), with a mean age of 8.78 years at Time 1 (SD = 1.03, age range = 7–10 years). There were no significant differences between the included and excluded children on the primary study variables [Trust: *t* (99) = −0.739, *p* = 0.462; Relationship closeness: *t* (99) = −0.020, *p* = 0.984; Reputation for trustworthiness: *t* (99) = −0.078, *p* = 0.938].

### Procedure

All assessments were conducted in participants’ classrooms with the assistance of two experimenters. In each of the three waves of the study, participants first reported on how much they trusted each of their classmates. Next, they reported on the closeness of their relationship with each of their classmates. Finally, they completed a sociometric assessment that was used as an index of each classmate’s reputation for trustworthiness. Each of these measures is described below.

### Measures

#### Interpersonal Trust

In order to assess trust, participants were given a list of all their classmates and were asked to respond to two questions about each one. The first question asked whether the participants are willing to let the classmate to take care of their possessions. The second question asked whether the participants are willing to share the classmate their secrets. Participants responded to these items on a 5-point scale that ranged from 1 (not at all) to 5 (extremely well). This assessment was developed with a separate sample of 63 Chinese elementary school children (33 girls, Mean of age = 9.87, SD = 1.52) asked what they would do to show they trusted someone. The results showed the two most frequently mentioned were to let the individual take care of the participant’s possessions (83% of children) and to share the individual the participant’s secrets (71% of children). The mean of these two ratings was computed to create an *Interpersonal Trust Score* for each classmate of each participant. The *taking care of possession* and *keeping secrets* scores were highly correlated with each other at each time point of rating (Time 1: *r* = 0.68, *p* < 0.001; Time 2: *r* = 0.64, *p* < 0.001; Time 3: *r* = 0.63, *p* < 0.001).

#### Relationship Closeness

Participants rated the same set of classmates as on the trust measure, but this time they rated the closeness of their relationship with each individual on 5-point scale that ranged from 1 (not at all close) to 5 (extremely close). These ratings were used to create a separate *Relationship Closeness Score* between each individual and every other individual in the class. Each relationship closeness score reflected the trustor’s rating of the trustee.

#### Reputation for Trustworthiness

A peer nomination method was used to assess the reputation of each participant. Three measures were included in this assessment: (1) how honest the target was perceived to be, (2) how well the target could be expected to keep his or her promise, and (3) how considerate the target was perceived to be (see Mayer et al., 1995). Specifically, research assistants evaluated those items and selected those that were judged to be most appropriate in a Chinese context.

The following illustrates the procedure, using the honesty measure as an example. Participants were given a list of classmates asked to indicate the three who were most honest and the three who were most dishonest. For each classmate, the number of negative nominations was then subtracted from the number of positive nominations to create a comprehensive honesty reputation score. To control the influence of different class sizes, we standardized these scores for each classmate in each class. The same procedure was followed with the other two reputation measures (promise-keeping and benevolence), and the means of these three standardized ratings were computed to create a *Reputation for Trustworthiness Score* for each classmate. The Cronbach’s coefficients for three items were high at each time point (Time 1: α = 0.93; Time 2: α = 0.93; Time 3: α = 0.94).

### The Plan of Analysis

First, we used SPSS13.0 to conduct descriptive analysis on each variable in this study, including the mean value, standard deviation of the variables and the correlation coefficient among them. Second, since the data in this study has a nested structure we would adopt Hierarchical Linear Model by using the HLM 6.08. Finally, we would present in two parts which were based on the cross-sectional and longitudinal data respectively.

## Results

### Cross-Sectional Data Analysis

#### Descriptive Statistics

Table 1 shows mean, standard deviation and correlations for the three assessments. As can be seen from this table, interpersonal trust scores were positively correlated with both relationship closeness scores and reputation for trustworthiness scores.

**TABLE 1:** Descriptive statistics and correlation between primary study variables of cross-sectional data (*N* = 178).

	Trust	Relationship	Reputation
**Descriptive statistics**			
Mean	2.56	2.52	0.00
SD	0.78	0.74	0.93
**Correlation**			
Trust		0.73***	0.42***
Relationship			0.29**

**p < 0.01, and ***p < 0.001.

#### Hierarchical Liner Modeling Results

Hierarchical Liner Modeling (HLM) is a multilevel modeling technique that deals with the inherent nested nature of data (Sripada and Rauch, 2015). Because the measurements were nested within the individual, we constructed a 2-levels model to assess the influence of relationship closeness and reputation for trustworthiness. HLM Version 6.08 was used for the HLM analysis.

##### Construction of the Final Model

Models of cross-sectional data were based on 7,421 measurements (Level-1) nested within 178 participants (Level-2). In the first step, the null model estimated components of variance for Level-1 and Level-2. The value of random effects of Level-2 (*τ_00_*) in this null model was significantly different from zero [*τ_00_* = 0.36, χ*^2^*(177) = 1,740.41, *p* < 0.001]. In addition, the intra-class correlation (ICC) for the individual level was 0.211, suggesting that 21.1% of variance in measurements could be accounted for by factors related to individuals. These results suggested that HLM analysis was needed (Cohen, 1988).

In second step, all the assessed trustee variables were included in the model. Specifically, trustee gender (male = 0, female = 1), relationship closeness scores, and reputation for trustworthiness scores were used as predictors of interpersonal trust Scores. After including variables of measurement, the model fit was improved [Δχ*^2^*(9) = 5,851.67, *p* < 0.001].

In third step, the final model was constructed with intercept and individual variables (age and gender of trustor) in Level-2 as following. In the cross-sectional data, the deviance of the null model is 25,417.40 (*df* = 2), the deviance of the final model is 19,553.46 (*df* = 11), so the new model explains 23.07% of the variation.

Level-1

Trust = π*_0_* + π*_1_* × trustee gender + π_2_ × relationship + π*_3_* × reputation + ε (1a)

Level-2

π*_0_* = β*_00_* + μ*_0_*                           (1b)
π*_1_* = β*_10_* + μ*_1_*                           (1c)
π*_2_* = β*_20_* + β*_21_* × trustor gender + β*_22_* × trustor age + μ*_2_*            (1d)
π*_3_* = β*_30_* + β*_31_* × trustor gender + β*_32_* × trustor age + μ*_3_*            (1e)

##### The Results of the Final Model

First, fixed effects on the final model (see Table 2) showed that relationship closeness, reputation for trustworthiness had significant effects on children’s interpersonal trust, with children relatively more trusting of trustees who were closer to them, and of trustees with a better reputation for trustworthiness. However, trustee gender had no significant effects, suggesting that children did not differ in their level of trust for girls vs. boys.

**TABLE 2:** Results of the final model of HLM of cross-sectional data.

Fixed effects:	β	SE	*t*
**Level-1**			
β*_00_*: intercept	2.57	0.05	54.41***
β*_10_*: trustee gender	0.08	0.05	1.61
β*_20_*: relationship	0.56	0.01	37.60***
β*_30_*: reputation	0.31	0.02	19.03***
**Level-2**			
β*_21_*^a^: relationship × trustor gender	0.08	0.03	2.54*
β*_22_*^b^: relationship × trustor age	0.06	0.01	5.84***
β*_31_*^c^: reputation × trustor gender	<0.01	0.03	0.17
β*_32_*^d^: reputation × trustor age	–0.04	0.01	−3.99***

*^*a*^β*_21_* was in formula (1d) and indicated the interaction between relationship and gender of trustor.*

*^*b*^β*_22_* was in formula (1d) and indicated the interaction between relationship and age of trustor.*

*^*c*^β*_31_* was in formula (1e) and indicated the interaction between reputation and gender of trustor.*

*^*d*^β*_32_* was in formula (1e) and indicated the interaction between reputation and age of trustor.*

*p < 0.05, and ***p < 0.001.

Second, the interaction between relationship closeness and trustor gender was significant, indicating that relationship closeness has a greater influence on interpersonal trust for girls than for boys. However, no interaction between reputation for trustworthiness and trustor gender was found, suggesting that the influences of trustworthiness on interpersonal trust are similar for girls and boys.

Finally, the interaction between relationship closeness and trustor age was positive and significant (β_22_ = 0.06, *p* < 0.001), indicating that the effect of relationship closeness on trust increased with trustor age. We also found that the interaction between reputation for trustworthiness and trustor age was negative and significant (β_32_ = −0.04, *p* = 0.001), which indicates that the effect of reputation for trustworthiness decreased with trustor age.

### Longitudinal Data Analysis

#### Descriptive Statistics

Table 3 shows the mean, standard deviation and correlations among the three wave measurements. As can be seen from the table, interpersonal trust scores were positively correlated with relationship closeness scores and with reputation for trustworthiness scores, both currently and longitudinally.

**TABLE 3:** Descriptive statistics and correlation between primary study variables in longitudinal data (*N* = 98).

		Time 1			Time 2			Time 3		
		Trust	Relationship	Reputation	Trust	Relationship	Reputation	Trust	Relationship	Reputation
**Descriptive statistics**										
	Mean	2.47	2.56	0.00	2.37	2.39	0.00	2.19	2.22	0.00
	SD	0.63	0.84	0.93	0.77	0.86	0.93	0.68	0.71	0.93
**Correlations**										
Time 1	Trust	1	0.67***	0.41***	0.47***	0.45***	0.41***	0.44***	0.42***	0.35***
	Relationship		1	0.30*	0.39***	0.39***	0.29*	0.38***	0.35***	0.26*
	Reputation			1	0.42***	0.32**	0.84***	0.36***	0.31*	0.74***
Time 2	Trust				1	0.75***	0.42***	0.56***	0.51***	0.40***
	Relationship					1	0.32**	0.50***	0.50***	0.29*
	Reputation						1	0.38***	0.32*	0.84***
Time 3	Trust							1	0.76***	0.39***
	Relationship								1	0.33**
	Reputation									1

*p < 0.05, **p < 0.01, and ***p < 0.001.

#### Hierarchical Liner Modeling Results

The structure of the longitudinal data had three levels. The measurements were nested within measured time, and then measured time was nested within individuals. We built a 3-level model to examine the influence of relationship closeness and reputation for trustworthiness on interpersonal trust as children developed.

Construction of the final model. Models of longitudinal data were based on 12,071 measurements (Level-1) nested within 271 measure times (Level-2), which were nested within 98 individuals (Level-3). First, the null model estimated the variance components for Level-1, Level-2 and Level-3. The value of the random effect of Level-2 (*τ*π_0_) and Level-3 (*τ*β_00_) was significant from zero [*τ*π_0_ = 0.22, χ^2^(173) = 1201.06, *p* < 0.001; *τ*β_00_ = 0.25, χ^2^(97) = 363.93, *p* < 0.001] and the ICC for measurements nested within measure times was 0.138, and for measure times nested within individuals was 0.157. These results suggested that HLM analysis was needed (Cohen, 1988).

In the second step, variables of Level-1 were used in the model, including trustee gender (male = 0; female = 1), relationship closeness scores, and reputation for trustworthiness scores, and significant decline of deviance suggested an improved model fit [Δχ^2^ (21) = 9,516.50, *p* < 0.001].

In the third step, time was included as variable in Level-2, and the first time point was coded as -1, the second as 0, and the third as 1. There was also a significant decline of deviance [Δχ2 (13) = 31.70, *p* < 0.05], indicating an improved model fit.

In the final step, trustor gender (male = 0; female = 1) and trustor age included in the model as variables of Level-3 and constructed the final model (shown below). In the longitudinal data, the deviance of the null model is 40,478.51 (*df* = 4), the deviance of the final model is 30,903.31 (*df* = 44), so the new model explains 23.66% of the variation.

Level-1

Trust = π_0_ + π_1_ × trustee gender + π_2_ × relationship + π_3_ × reputation + ε (2a)

Level-2

π_0_ = β_00_ + μ_0_                         (2b)
π_1_ = β_10_ + μ_1_                         (2c)
π_2_ = β_20_ + β_21_ × time + μ_2_                      (2d)
π_3_ = β_30_ + β_31_ × time + μ_3_                      (2e)

Level-3

β_00_ = γ_000_ + *e*_00_                           (2f)
β_10_ = γ_100_ + *e*_10_                           (2g)
β_20_ = γ_200_ + γ_201_ × trustor gender + γ_202_ × trustor age + *e*_20_          (2h)
β_21_ = γ_210_ + γ_211_ × trustor age + *e*_21_                    (2i)
β_30_ = γ_300_ + γ_301_ × trustor gender + γ_302_ × trustor age + *e*_30_          (2j)
β_31_ = γ_310_ + γ_311_ × trustor age + *e*_31_                   (2k)

The results of the final model. First, fixed effects on the final model (see Table 4) showed that relationship closeness scores, reputation for trustworthiness scores had a significant positive effect on children’s interpersonal trust scores. Specifically, children were more likely to trust classmates who were closer to them, and classmates who were rated as having a reputation for being trustworthy. But, the gender of trustee had no significant effect, suggesting children equally trust girls and boys.

**TABLE 4:** Results of the final model of HLM in longitudinal data.

Fixed effects:	β	*SE*	*t*
**Level-1**			
γ*_000_*: intercept	2.31	0.06	36.05***
γ*_100_*: trustee gender	0.06	0.04	1.67
γ*_200_*: relationship	0.47	0.03	17.85***
γ*_300_*: reputation	0.26	0.02	11.17***
**Level-2**			
γ*_210_*^a^: relationship × time	0.03	0.01	2.32*
γ*_310_*^b^: reputation × time	–0.05	0.01	−4.719***
**Level-3**			
γ*_201_*^c^: relationship × trustor gender	0.13	0.03	3.81***
γ_202_^d^: relationship × trustor age	0.05	0.01	3.40***
γ*_211_*^e^: relationship × time × trustor age	–0.03	0.01	−2.34*
γ*_301_*^f^: reputation × trustor gender	<0.01	0.03	0.24
γ*_302_*^g^: reputation × trustor age	–0.04	0.01	−2.84**
γ*_311_*^h^: reputation × time × trustor age	0.02	0.01	2.32*

*^*a*^γ*_210_* was in formula (2d) and indicated the interaction between relationship and time.*

*^*b*^γ*_310_* was in formula (2e) and indicated the interaction between reputation and time.*

*^*c*^γ*_201_* was in formula (2h) and indicated the interaction between relationship and gender of trustor.*

*^*d*^γ*_202_* was in formula (2h) and indicated the interaction between relationship and trustor age.*

*^*e*^γ*_211_* was in formula (2i) and indicated the interaction between relationship and time and trustor age.*

*^*f*^γ*_301_* was in formula (2j) and indicated the interaction between reputation and trustor gender.*

*^*g*^γ*_302_* was in formula (2j) and indicated the interaction between reputation and age of trustor.*

*^*h*^γ*_311_* was in formula (2k) and indicated the interaction between reputation and time and trustor age.*

*p < 0.05, **p < 0.01, and ***p < 0.001.

**FIGURE 1:**
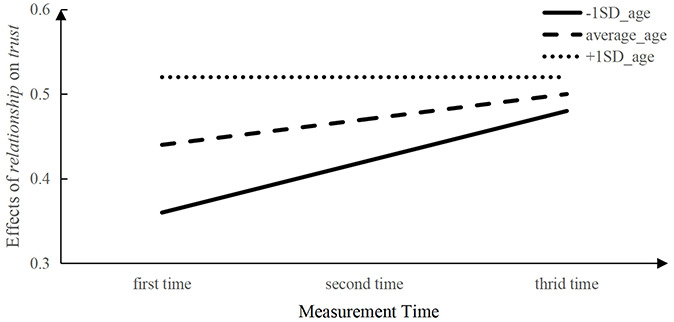
A simple slops analysis of the effect of *relationship* on children’s *trust*.

Second, the interaction between relationship closeness scores and time was significant and positive (γ_210_ = 0.03, *p* = 0.023). These results indicated that the effect of relationship closeness on children’s interpersonal trust increased with age, which was consistent to what was observed in the cross-sectional data. We also found the moderating effect of trustor age on the relation between relationship closeness and time was significant (γ_211_ = −0.03, *p* < 0.05). In order to understand the moderating effect of trustor’s age more clearly, we took *measurement time* as the horizontal axis and *effects of relationship closeness on trust* as the vertical axis, and then separated the initial trustor age into three levels as average_age, +1 SD_age and + 1 SD_age for plotting. As shown in Figure 1, we found the effect sizes of relationship closeness on trust were increased with measure time generally, indicating that relationship plays an increasingly important role in children’s trust judgments with age. In addition, the initial age of children had a moderating effect on the effect of relationship closeness on children’s trust, the younger children had steeper increasing curve than older children.

**FIGURE 2:**
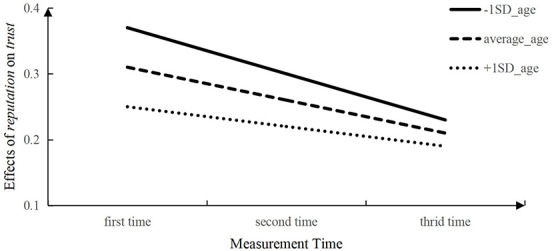
A simple slopes analysis of the effect of *reputation* on children’s *trust*.

Third, the interaction between reputation for trustworthiness scores and time was significant and negative (γ_310_ = −0.05, *p* < 0.001). These results indicated that the effect of reputation for trustworthiness on interpersonal trust decreased with age, which were consistent to what was observed in the cross-sectional data. We also found the moderating effect of trustor age on the interaction between relationship closeness and time was significant (γ_311_ = 0.02, *p* < 0.05). Similarity, we took *measurement time* as the horizontal axis and *effects of reputation for trustworthiness on trust* as the vertical axis, and then separated the initial trustor age into three levels as average_age, + 1 SD_age and + 1 SD_age for plotting. As shown in Figure 2, we found the effect sizes of reputation for trustworthiness on trust were decreased with measure time generally, indicating that reputation played a decreasingly important role in children’s trust judgments with age. In addition, the initial age of children had a moderating effect on the effect of reputation for trustworthiness on children’s trust: younger children has steeper decreasing curve than older children.

Finally, as is consistent with the results of cross-sectional data, we found a significant interaction between relationship closeness scores and trustor gender (γ_201_ = 0.13, *p* < 0.001) again, indicating that relationship closeness had a greater effect on interpersonal trust for girls than for boys. There was no significant interaction between reputation for trustworthiness and trustor gender.

## Discussion

The present research examined how children’s trust develops over time within the context of social interactions (Lewicki et al., 2006). We did so through the lens of theoretical frameworks that conceptualize trust in terms of a willingness to make oneself vulnerable to others (Ring and Van de Ven, 1992; Sitkin and Roth, 1993; Mayer et al., 1995; Hall et al., 2001; Bos et al., 2002). Chinese children between the ages of 7 and 13 were tested using a cross-sectional research design, and a subset of these children was tested using a longitudinal research design over a period of about 2 years. Both the cross-sectional and the longitudinal results suggest that children first learn to trust based on the extent to which potential trustees are generally trustworthy, and later begin to focus on the nature of their relationship closeness with the trustee.

These findings are consistent with prior research indicating that when children across a wide range of ages make trust judgments they take into account what is generally known about the potential trustee as well as the nature of their relationship closeness with potential trustee (Betts and Rotenberg, 2008; Betts et al., 2014). Our work demonstrates for the first time that children’s use of these cues changes as they develop, and it provides the first evidence that with development, children’s trust increasingly reflects the nature of their relationship with the individual in question.

What is the cause of this pattern of developmental change? One possibility is that the more time individuals spend together, the more relevant experience trustors have to draw upon when deciding whether a particular trustee can be counted on in situations that involve personally meaningful risks. For example, trustors may consider previous experiences in which they have counted on specific trustees to assess their likelihood of doing so in the future, much like young children use an informant’s prior history of reliability to determine whether he or she is likely to be a good source of factual information (Pasquini et al., 2007).

Another possibility is that children become increasingly accurate at judging trustworthiness with development and eventually come to recognize that behaviors and expectations that are related to trustworthiness are likely to be different within the context of close relationships (see Kahn and Turiel, 1988). For example, it would not be surprising if children take special care of possessions owned by their close friends. This might be the case because they are likely to have a higher level of genuine concern for their close friends or because there would be more at stake if a close friend’s possession were lost. It may also be that acts of loyalty serve as a primary indicator of trust, and that a willingness to show loyalty tends to be highly relationship-specific.

We also found that relationship closeness played a stronger role in girls’ interpersonal trust evaluations than it did in boys’ interpersonal trust evaluations. This finding is consistent with previous research suggesting that girls may prioritize relationship closeness more than boys (Benenson and Schinazi, 2004; Watson, 2012). From the evolutionary perspective, males tend to be more self-focused, females tend to converse more about others and tend to focus more on building and maintaining social networks. But males tend to focus more on display and status. In addition, a meta-analysis (Hall, 2011) found two major gender differences in friendships: communion is higher in females, agency is higher in males. Communion refers to the intimacy or closeness needs that are met through friendship (Watson, 2012), and the agency element of friendship provides individuation and power needs. In children’s trust evaluations, they may need to communicate and familiarize themselves with each other to establish intimacy and enhance interpersonal closeness, and girls seem to be better at this.

Further research will also be needed to assess some limitations in our work. First, it will also be important to examine how culturally specific vs. general our findings are. One challenge in doing so is that we specifically developed measures to fit a Chinese context. Because based our research on the Chinese conceptualization of trust as being highly relationship-specific (Peng, 1998), it is plausible that our findings of age-related changes may be culturally specific. Additionally, it is also possible that the collectivist values and an associated focus on social obligations (Oyserman et al., 2002), might promote a focus on the importance of relationship specific trust and the understanding that trust is closely tied to reciprocity. However, it is also plausible that our findings are generalizable, as is suggested by evidence that children’s dyadic relationships have important psychological and social implications in Western societies as well (Berndt, 2002; Bagwell and Schmidt, 2011).

A related issue is that we cannot assess the effects of the widespread practice in China of grouping children with the same set of classmates over multiple years. All participants in our study were grouped in this way, meaning that the age-related changes we observed may be dependent on close observation of the same individuals over an extended period of time. This emphasizes the need to examine the extent to which our findings will generalize across cultures. It is possible that the findings are generalizable in the sense that once children know each other well, trust becomes more relationship-specific.

Finally, although we designed an item and conceptual distinction between interpersonal trust and interpersonal relationship, to some extent, trust is still highly correlated with interpersonal relationships which implied the common method bias. In addition, some constructs have been investigated by using a small number of items or questions as well as lack of using different informants is also a limitation of this study.

To conclude, this study highlights the distinction between trusting someone because of what they are generally like, vs. what they are like within the context of a specific interpersonal relationship. Our findings suggest that as children get older their sense of how trustworthy individuals are becomes increasingly linked to their relationship closeness.

## Data Availability Statement

The raw data supporting the conclusions of this article will be made available by the authors, without undue reservation.

## Ethics Statement

The studies involving human participants were reviewed and approved by University Research Ethics Committee. Written informed consent to participate in this study was provided by the participants’ legal guardian/next of kin.

## Author Contributions

QL: experiment design and draft writing. ZL: data collection and draft writing. WZ: data collection. YW: data analysis. GH: research conception and experiment design. All authors contributed to the article and approved the submitted version.

## Conflict of Interest

The authors declare that the research was conducted in the absence of any commercial or financial relationships that could be construed as a potential conflict of interest.

## Publisher’s Note

All claims expressed in this article are solely those of the authors and do not necessarily represent those of their affiliated organizations, or those of the publisher, the editors and the reviewers. Any product that may be evaluated in this article, or claim that may be made by its manufacturer, is not guaranteed or endorsed by the publisher.

## References

[B1] Bagwell C. L. Schmidt M. E. (2011). The friendship quality of overtly and relationally victimized children. *Merrill-Palmer Q.* 57 158–185. 10.1353/mpq.2011.0009 34409987

[B2] Benenson J. F. Schinazi J. (2004). Sex differences in reactions to outperforming same-sex friends. *Br. J. Dev. Psychol.* 22 317–333. 10.1348/0261510041552729

[B3] Berndt T. J. (2002). Friendship quality and social development. *Curr. Dir. Psychol. Sci.* 11 7–10. 10.1111/1467-8721.00157

[B4] Berndt T. J. Perry T. B. (1986). Children’s perception of friendships as supportive relationships. *Dev. Psychol.* 22 640–648. 10.1037/0012-1649.22.5.640

[B5] Betts L. R. Rotenberg K. J. (2008). A social relations analysis of children’s trust in their peers across the early years of school. *Soc. Dev.* 17 1039–1055. 10.1111/j.1467-9507.2008.00479.x

[B6] Betts L. R. Rotenberg K. J. Petrocchi S. Lecciso F. Sakai A. Maeshiro K. (2014). An investigation of children’s peer trust across culture: is the composition of peer trust universal? *Int. J. Behav. Dev.* 38 33–41. 10.1177/0165025413505248

[B7] Birch S. A. J. Vauthier S. A. Bloom P. (2008). Three- and four-year-olds spontaneously use others’ past performance to guide their learning. *Cognition* 107 1018–1034. 10.1016/j.cognition.2007.12.008 18295193

[B8] Bos N. Olson J. Gergle D. Olson G. Wright Z. (2002). Effects of four computer-mediated communications channels on trust development. *Paper Presented at the CHI 2002 Conference on Human Factors in Computing Systems: Changing Our World, Changing Ourselves*, Minneapolis MIN, 10.1145/503376.503401

[B9] Boseovski J. J. Thurman S. L. (2014). Evaluating and approaching a strange animal: children’s trust in informant testimony. *Child Dev.* 85 824–845. 10.1111/cdev.12156 24032359

[B10] Cohen J. (1988). *Statistical Power Analysis for the Behavioral Science.* Mahwah, NJ: L. Erlbaum Associates.

[B11] Colquitt J. A. Scott B. A. LePine J. A. (2007). Trust, trustworthiness, and trust propensity: a meta-analytic test of their unique relationships with risk taking and job performance. *J. Appl. Psychol.* 92 909–927. 10.1037/0021-9010.92.4.909 17638454

[B12] Corriveau K. Harris P. L. (2009). Preschoolers continue to trust a more accurate informant 1 week after exposure to accuracy information. *Dev. Sci.* 12 188–193. 10.1111/j.1467-7687.2008.00763.x 19120427

[B13] Corriveau K. H. Fusaro M. Harris P. L. (2009). Going with the flow: preschoolers prefer non-dissenters as informants. *Psychol. Sci.* 20 372–377. 10.1111/j.1467-9280.2009.02291.x 19207691

[B14] Damon W. (1983). *Social and Personality Development: Infancy Through Adolescence.* New York, NY: W. W. Norton.

[B15] Deutsch M. (1958). Trust and suspicion. *J. Conflict Resolut.* 2 265–279. 10.1177/002200275800200401

[B16] Fu G. Heyman G. D. Chen G. Liu P. Lee K. (2015). Children trust people who lie to benefit others. *J. Exp. Child Psychol.* 129 127–139. 10.1016/j.jecp.2014.09.006 25443139

[B17] Gelman S. A. (2009). Learning from others: children’s construction of concepts. *Annu. Rev. Psychol.* 60 115–140. 10.1146/annurev.psych.59.103006.093659 18631027 PMC2829654

[B18] Hall J. A. (2011). Sex differences in friendship expectations: a meta-analysis. *J. Soc. Pers. Relat.* 28 723–747. 10.1177/0265407510386192

[B19] Hall M. A. Dugan E. Zheng B. Mishra A. K. (2001). Trust in physicians and medical institutions: what is it, can it be measured, and does it matter? *Milbank Q.* 79 613–639. 10.1111/1468-0009.00223 11789119 PMC2751209

[B20] Harris P. L. (2007). Trust. *Dev. Sci.* 10 135–138. 10.1111/j.1467-7687.2007.00575.x 17181711

[B21] Hartup W. W. (1996). The company they keep: friendships and their developmental significance. *Child Dev.* 67 1–13. 10.1111/j.1467-8624.1996.tb01714.x 8605821

[B22] Heyman G. D. (2008). Children’s critical thinking when learning from others. *Curr. Dir. Psychol. Sci.* 17 344–347. 10.1111/j.1467-8721.2008.00603.x 20936054 PMC2951681

[B23] Jaswal V. K. Neely L. A. (2006). Adults don’t always know best: preschoolers use past reliability over age when learning new words. *Psychol. Sci.* 17 757–758. 10.1111/j.1467-9280.2006.01778.x 16984291

[B24] Johnson-George C. L. Swap W. C. (1982). The measurement of specific interpersonal trust: construction and validation of a scale to assess trust in a specific other. *J. Pers. Soc. Psychol.* 43 1306–1317. 10.1037/0022-3514.43.6.1306

[B25] Kahn P. Turiel E. (1988). Children’s conceptions of trust in the context of social expectations. *Merrill-Palmer Q.* 34 403–419. 10.1007/BF01537885 24277670

[B26] Koenig M. A. Harris P. L. (2005). Preschoolers mistrust ignorant and inaccurate speakers. *Child Dev.* 76 1261–1277. 10.1111/j.1467-8624.2005.00849.x 16274439

[B27] Landrum A. R. Mills C. M. Johnston A. M. (2013). When do children trust the expert? Benevolence information influences children’s trust more than expertise. *Dev. Sci.* 16 622–638. 10.1111/desc.12059 23786479

[B28] Lane J. D. Harris P. L. (2015). The roles of intuition and informants’ expertise in children’s epistemic trust. *Child Dev.* 86 919–926. 10.1111/cdev.12324 25425347 PMC4428962

[B29] Lane J. D. Wellman H. M. Gelman S. A. (2013). Informants’ traits weigh heavily in young children’s trust in testimony and in their epistemic inferences. *Child Dev.* 84 1253–1268. 10.1111/cdev.12029 23240893 PMC3601569

[B30] Lewicki R. J. Bunker B. B. (1995). “Trust in relationships: a model of development and decline,” in *Conflict, Cooperation and Justice: Essays Inspired by the Work of Morton Deutsch*, eds Bunker B. B. Rubin J. Z. (San Francisco, CA: Jossey-Bass), 133–173.

[B31] Lewicki R. J. Tomlinson E. C. Gillespie N. (2006). Models of interpersonal trust development: theoretical approaches, empirical evidence, and future directions. *J. Manag.* 32 991–1022. 10.1177/0149206306294405

[B32] Li Q. G. Heyman G. D. Xu F. Lee K. (2014). Young children’s use of honesty as a basis for selective trust. *J. Exp. Child Psychol.* 117 59–72. 10.1016/j.jecp.2013.09.002 24149377

[B33] Mayer R. C. Davis J. H. Schoorman F. D. (1995). An integrative model of organizational trust. *Acad. Manag. Rev.* 20 709–734. 10.5465/AMR.1995.9508080335

[B34] Mcauley C. Mckeown C. Merriman B. (2012). Spending time with family and friends: children’s views on relationships and shared activities. *Child Indic. Res.* 5 449–467. 10.1007/s12187-012-9158-2

[B35] Mills C. M. (2013). Knowing when to doubt: developing a critical stance when learning from others. *Dev. Psychol.* 49 404–425. 10.1037/a0029500 22889395 PMC3810952

[B36] Oberle E. Schonert-Reichl K. A. Thomson K. C. (2010). Understanding the link between social and emotional well-being and peer relations in early adolescence: gender-specific predictors of peer acceptance. *J. Youth Adolesc.* 39 1330–1342. 10.1007/s10964-009-9486-9 20091211

[B37] Oyserman D. Coon H. Kemmelmeier M. (2002). Rethinking individualism and collectivism: evaluation of theoretical assumptions and meta-analyses. *Psychol. Bull.* 128 3–73. 10.1037/0033-2909.128.1.3 11843547

[B38] Pasquini E. S. Corriveau K. H. Koenig M. Harris P. L. (2007). Preschoolers monitor the relative accuracy of informants. *Dev. Psychol.* 43 1216–1226. 10.1037/0012-1649.43.5.1216 17723046

[B39] Peng S. (1998). *Guanxi in Trust: An Indigenous Study of Chinese Interpersonal trust.* Ph. D. Thesis. Pokfulam: University of Hong Kong, 10.5353/th_b3123762

[B40] Poulin-Dubois D. Chow V. (2009). The effect of a looker’s past reliability on infants’ reasoning about beliefs. *Dev. Psychol.* 45 1576–1582. 10.1037/a0016715 19899915

[B41] Rapp D. J. Engelmann J. M. Herrmann E. Tomasello M. (2019). Young children’s reputational strategies in a peer group context. *Dev. Psychol.* 55 329–345. 10.1037/dev0000639 30525833

[B42] Ring P. S. Van de Ven A. (1992). Structuring cooperative relationships between organizations. *Strateg. Manag. J.* 13 483–498. 10.1002/smj.4250130702

[B43] Rotenberg K. J. McDougall P. Boulton M. J. Vallancourt T. Fox C. Hymel S. (2004). Cross sectional relations among trustworthiness, social relationships, and psychological adjustment in children and early adolescents from the United Kingdom and Canada. *J. Exp. Child Psychol.* 88 46–67. 10.1016/j.jecp.2004.01.005 15093725

[B44] Rotenberg K. J. Michalik N. Eisenberg N. Betts L. R. (2008). The relations among young children’s peer-reported trustworthiness, inhibitory control, and preschool adjustment. *Early Child. Res. Q.* 23 288–298. 10.1016/j.ecresq.2007.04.003 18846246 PMC2563798

[B45] Shaw A. Montinari N. Piovesan M. Olson K. R. Gino F. Norton M. I. (2014). Children develop a veil of fairness. *J. Exp. Psychol. Gen.* 143 363–375. 10.1037/a003124 23317084

[B46] Sitkin S. B. Roth N. L. (1993). Explaining the limited effectiveness of legalistic “remedies” for trust/distrust. *Organ. Sci.* 4 367–392. 10.1287/orsc.4.3.367 19642375

[B47] Sripada R. K. Rauch S. A. (2015). Between-session and within-session habituation in prolonged exposure therapy for posttraumatic stress disorder: a hierarchical linear modeling approach. *J. Anxiety Disord.* 30 81–87. 10.1016/j.janxdis.2015.01.002 25613235

[B48] Taylor M. G. (2013). Gender influences on children’s selective trust of adult testimony. *J. Exp. Child Psychol.* 115 672–690. 10.1016/j.jecp.2013.04.003 23708732

[B49] Tomasello M. Hamann K. (2012). Collaboration in young children. *Q. J. Exp. Psychol.* 65 1–12. 10.1080/17470218.2011.608853 22171893

[B50] Watson D. C. (2012). Gender differences in gossip and friendship. *Sex Roles* 67 9–19. 10.1007/s11199-012-0160-4

[B51] Xu F. Evans A. D. Li C. Li Q. Heyman G. Lee K. (2013). The role of honesty and benevolence in children’s judgments of trustworthiness. *Int. J. Behav. Dev.* 37 257–265. 10.1177/0165025413479861

