# Supplementary material for: Cross-Sectional and Longitudinal Associations Among Children’s Interpersonal Trust, Reputation for Trustworthiness, and Relationship Closeness
Source: Front Psychol. 2021 Oct 1;12:634540. doi: 10.3389/fpsyg.2021.634540 (PMC8517192; doi:10.3389/fpsyg.2021.634540)
Supplement: Supplementary file 1 [file Data_Sheet_1.docx]

Supplementary Material

By its own setting, HLM software cannot output or edit its syntax like SPSS. But according to the HLM usage guidelines, to run an analysis in HLM, four steps are required:

(1) The type of model to be fitted must be decided on.

The HLM program encompasses 5 modules(but in this study, only HLM2＆HLM3 were used) that may be used to fit different types of models:

The HLM2 module is used to fit two-level linear and non-linear (HGLM) models. It offers the widest array of special features, output, and hypothesis testing options. The HLM3 module is used to fit three-level linear and non-linear (HGLM) models. The range of features are similar to that of HLM2.

(2) An appropriate MDM(Multivariate Data Matrix) file must be created.

The first task in using HLM is to construct the Multivariate Data Matrix (MDM) from raw data or from a statistical package.

Data file(s) must be sorted by the level-2 and, if using HLM3 or HMLM2, the level-3 ID. When creating an MDM file for HCM2, row and column IDs are needed. While it is possible to build the MDM file from a single data file, this option is not suggested when the data file is very large. Information on the [rules ID variables have conform to](mk:@MSITStore:D:\Desktop\研究生\HLM608\HLM608Trial\whlm.chm::/WHLM/Creating_MDM_files/Rules_for_ID_variables.htm), and the [construction of format statements when ASCII files are used](mk:@MSITStore:D:\Desktop\研究生\HLM608\HLM608Trial\whlm.chm::/WHLM/Creating_MDM_files/Rules_for_format_statements.htm) as input are given elsewhere.

(3) The model is specified and various statistical and output options specified.

Basic model specification has three steps:

①Specifying the level-1 model, which defines a set of level-1 coefficients to be computed for each level-2 unit.

②Specifying a level-2 structural model to predict each of the level-1 coefficients.

③Specifying the level-1 coefficients to be viewed as random or non-random.

After these three steps have been completed, a linear model is obtained.

(4) The model is run, after which model-based graphs can be obtained.

**In order to show the construction of the model more clearly, Here are the models(The Cross-sectional & The Longitudinal) we built in HLM:**

**The Cross-sectional:**


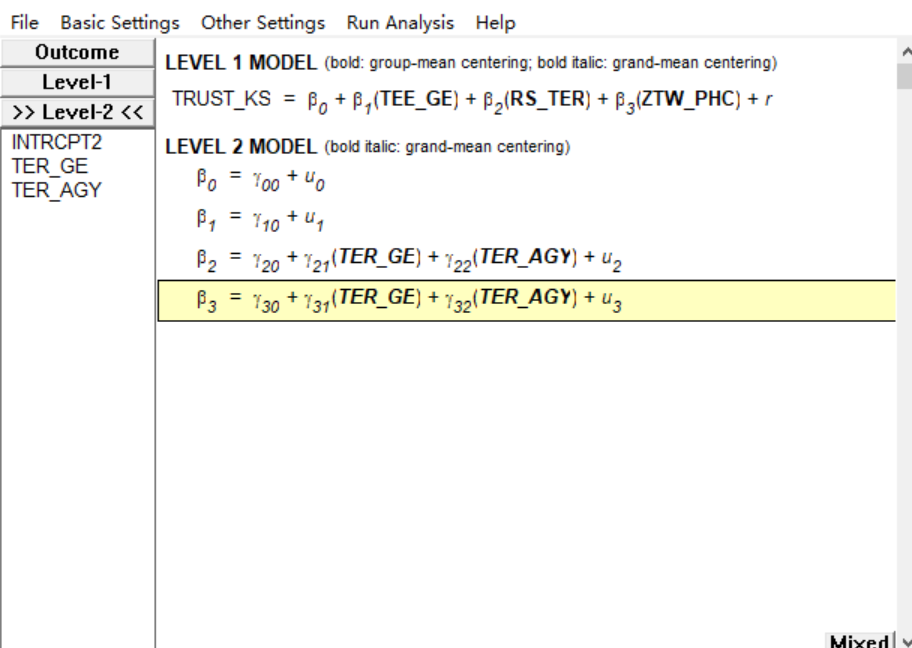


**FIGURE 1** *Note:* TEE_GE: trustee gender; RS_ter: relationship closeness; ZTW_PHC: reputation for trustworthiness; TER_GE: trustor gender; TER_AGY: age

The output results are as follows：


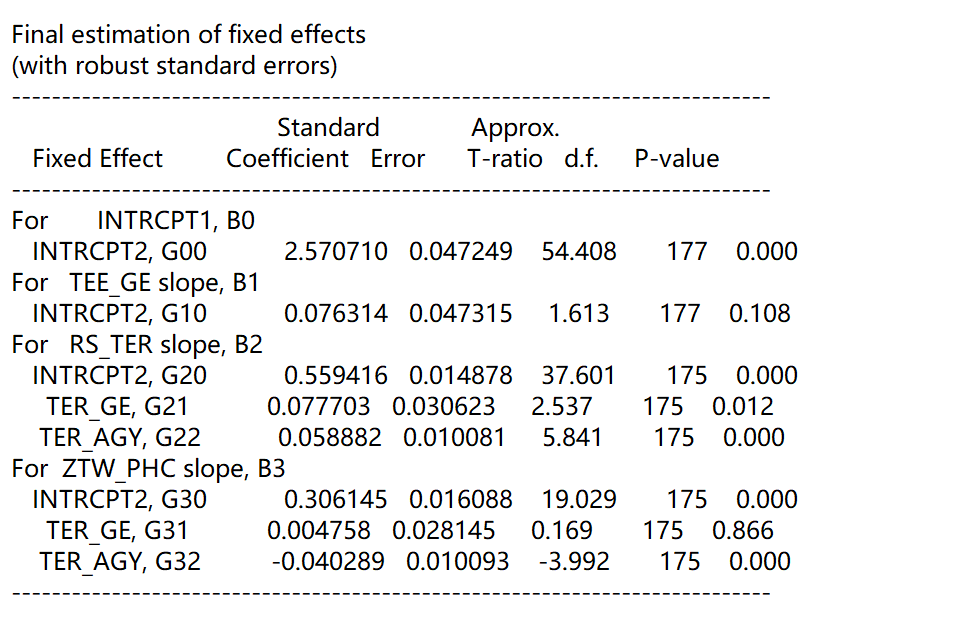
 **FIGURE 2**

**The Longitudinal:**

The model construction:


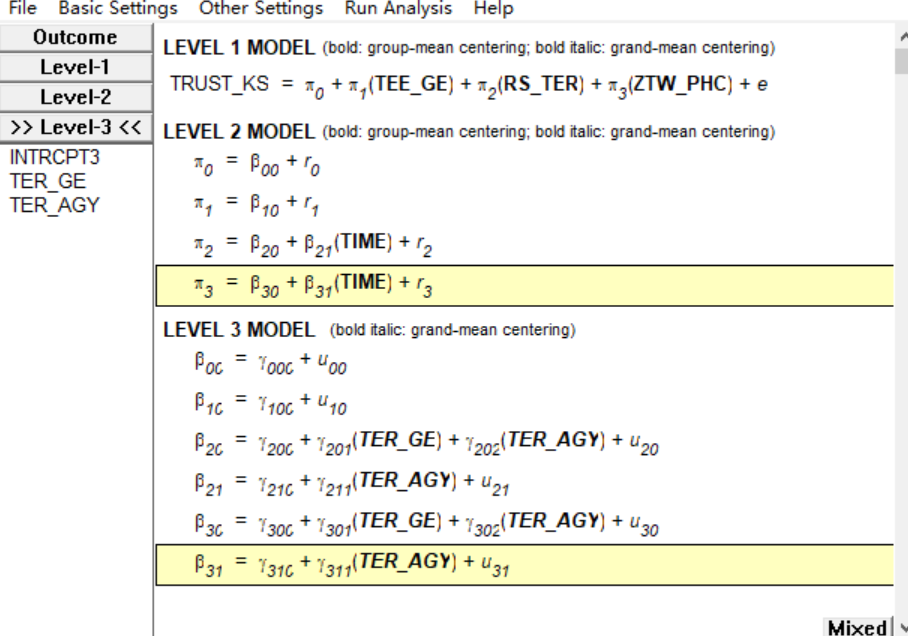


**FIGURE 3** *Note:* TEE_GE: trustee gender; RS_ter: relationship closeness; ZTW_PHC: reputation for trustworthiness; TIME: measuring time; TER_GE: trustor gender; TER_AGY: age

The output results are as follows：


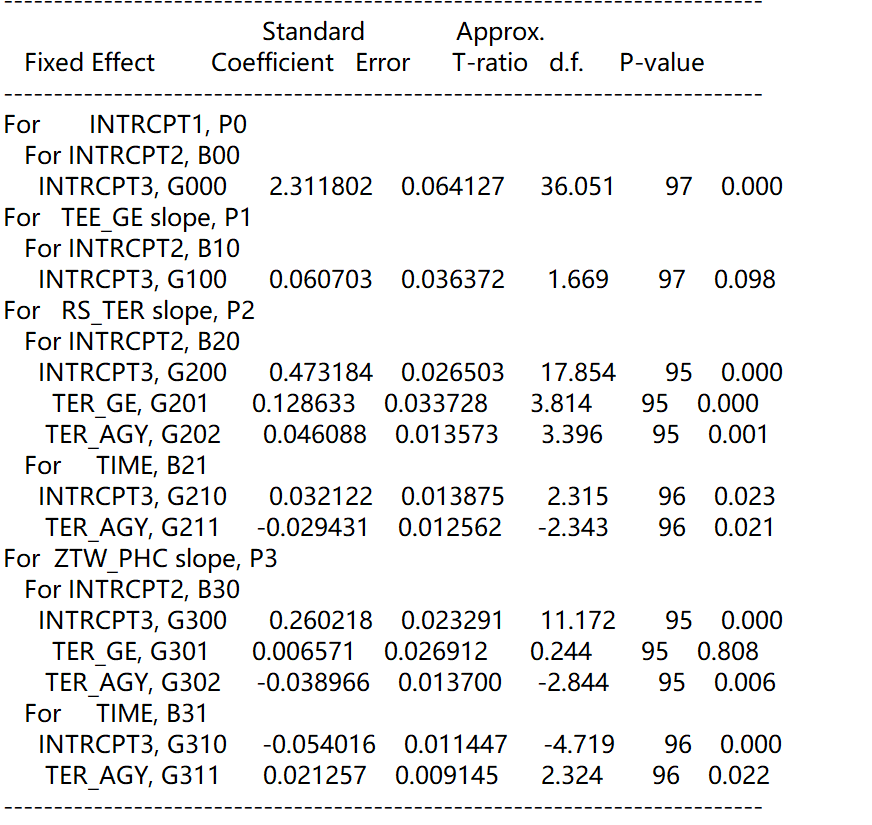


**FIGURE 4**
